# A comparative study on gene-set analysis methods for assessing differential expression associated with the survival phenotype

**DOI:** 10.1186/1471-2105-12-377

**Published:** 2011-09-26

**Authors:** Seungyeoun Lee, Jinheum Kim, Sunho Lee

**Affiliations:** 1 Department of Mathematics and Statistics, Sejong University, Seoul, 143-747, Korea; 2 Department of Applied Statistics, University of Suwon, Hwaseong, 445-743, Korea

## Abstract

**Background:**

Many gene-set analysis methods have been previously proposed and compared through simulation studies and analysis of real datasets for binary phenotypes. We focused on the survival phenotype and compared the performances of Gene Set Enrichment Analysis (GSEA), Global Test (GT), Wald-type Test (WT) and Global Boost Test (GBST) methods in a simulation study and on two ovarian cancer data sets. We considered two versions of GSEA by allowing different weights: GSEA1 uses equal weights, yielding results similar to the Kolmogorov-Smirnov test; while GSEA2's weights are based on the correlation between genes and the phenotype.

**Results:**

We compared GSEA1, GSEA2, GT, WT and GBST in a simulation study with various settings for the correlation structure of the genes and the association parameter between the survival outcome and the genes. Simulation results indicated that GT, WT and GBST consistently have higher power than GSEA1 and GSEA2 across all scenarios. However, the power of the five tests depends on the combination of correlation structure and association parameter. For the ovarian cancer data set, using the FDR threshold of *q *< 0.1, the GT, WT and GBST detected 12, 6 and 8 significant pathways, respectively, whereas neither GSEA1 nor GSEA2 detected any significant pathways. In addition, among the pathways found significant by GT, WT, and GBST, three pathways - Purine metabolism, Leukocyte transendothelial migration and Jak-STAT signaling pathway - overlapped with those reported in previous ovarian cancer microarray studies.

**Conclusion:**

Simulation studies and a real data example indicate that GT, WT and GBST tend to have high power, whereas GSEA1 and GSEA2 have lower power. We also found that the power of the five tests is much higher when genes are correlated than when genes are independent, when survival is positively associated with genes. It seems that there is a synergistic effect in detecting significant gene sets when significant genes have within-class correlation and the association between survival and genes is positive or negative (i.e., one-direction correlation).

## Background

Gene-set analysis is a microarray data analysis method that uses existing knowledge of biological pathways, or sets of individual genes that are linked via related biological functions. Gene-set analysis mainly aims to discover gene sets for which expression is associated with a phenotype of interest. Compared to single-gene analyses, gene-set analyses may lead to more interpretable results by yielding insights into biological mechanisms. Furthermore, considering gene sets rather than single genes can reduce problems associated with multiple testing, since there are typically far fewer gene pathways than individual genes. Initially, gene-set analyses focused on identifying biological pathways (i.e., sets of genes) that are differentially expressed between two classes of a phenotype such as tumor vs. normal cells. Mootha et al. [1] proposed Gene Set Enrichment Analysis (GSEA), based on the Kolmogorov-Smirnov statistic, which measures the maximum degree of differential gene expression in a gene set across a binary phenotype. Subramanian et al. [2] improved GSEA by weighting each gene according to its correlation with the phenotype, and calculating a running-sum statistic; in contrast, the original GSEA uses equal weights regardless of the correlation between genes and the phenotype. GSEA calculates a p-value by permuting the original data set, which is computationally intensive for a large dataset. Kim and Volsky [3] proposed a parametric analysis of gene-set enrichment, which calculates a Z-score for a given gene set from a parameter such as fold change value between two classes of a phenotype, and makes statistical inference from the asymptotic normal distribution of the Z score. Dinu et al. [4] described some critical problems with GSEA, and proposed an alternative method by extending an individual gene analysis method, Significance Analysis of Microarrays, to gene-set analysis (SAM-GS). In addition, they compared SAM-GS to GSEA using a mouse microarray dataset with simulated gene sets, and showed an advantage to SAM-GS over GSEA in the analysis of three real microarray datasets.

Recently, gene-set analysis methods have been expanded to include other kinds of phenotypes, such as censored survival time and quantitative traits. Goeman et al. [5] proposed the Global Test, a score statistic based on random-effects modeling of parameters corresponding to the coefficients of the individual genes in the pathway. Goeman et al. [6] extended the Global Test to assess association of a set of genes with survival time based on a Cox proportional hazards model. In addition, Binder and Schumacher [7] considered fitting high-dimensional survival models while allowing for mandatory clinical covariates using a boosting algorithm. Boulesteix and Hothorn [8] instead proposed the Global Boost Test, which combines a Cox model for modeling the clinical covariates with a boosting algorithm for modeling the additional predictive value of high-dimensional gene expression data. Furthermore, Adewale et al. [9] proposed a unified general analysis method for microarray data for identifying pathways whose expression is associated with a phenotype of any kind, adjusting for covariates that may also be associated with the phenotype of interest. This unified pathway analysis method combines the regression-based test statistic for each individual gene in a pathway of interest into a real pathway-level test statistic. The form of the test statistic is a sum of squares of the Wald statistic for individual genes in the pathway of interest.

Gene expression profiles have been used extensively in the prediction of tumor subtypes or patient survival (Alizadeh et al. [10], Golub et al. [11], and Rosenwald et al. [12]). Initially, many studies focused on expression levels of single genes to predict tumor subtype or patient survival. Among many thousands of microarray measurements, each relating to the expression level of a single gene, a subset of significant genes can be identified by constructing a prediction model using the lasso (Gui and Li [13], Tibshirani [14]), principal components analysis (Tian et al. [15]), supervised principal component analysis (Bair and Tibshirani [16]), support vector machines (Furey et al. [17]), and other methods. However, single genes are often not of primary interest because the activities of entire pathways or genomic regions that are suspected to be more biologically relevant. In addition, combining gene expression data with prior biological knowledge of groups of genes improves prediction accuracy and interpretability of survival models. The prediction of patient survival may be improved by integrating gene expression data with prior biological knowledge such as gene sets and pathways, as well as by adjusting for covariates such as age, sex, and other clinical variables. Chen and Wang [18] proposed a general strategy for improving prediction accuracy and interpretability by constructing pathway-based prediction models for survival, which outperform the prediction models based on expression levels of single genes.

Recently, Liu et al. [19] compared the statistical performance of three gene-set analysis methods - the Global Test, ANCOVA Global Test, and SAM-GS - for a binary phenotype based on simulated data and real microarray datasets. They reported similar performances for all three methods after appropriate standardization, given the use of permutation-based inference. They also showed the advantage of the Global Test and ANCOVA Global Test, which are able to analyze survival phenotypes and adjust for covariates. To our knowledge, however, the performances of gene-set analysis methods with a survival phenotype have rarely been studied via simulation. In this paper, we compare the performances of five gene-set analysis tests: two different GSEA tests(GSEA1 and GSEA2) [1,2], Global Test (GT) [6], Wald-type Test (WT) [9] and Global Boost Test (GBST) [8] for assessing differential expression associated with the survival phenotype based on a simulation dataset and a real dataset of ovarian cancer patients.

## Results

### Simulation experiment

We generated a simulation dataset by the following procedure to evaluate the performance of five gene-set analysis tests:

(i) We randomly generated observations from a multivariate normal with a zero-mean vector and a variance-covariance matrix ∑, denoted by *MVN*(0,∑).

(ii) We randomly generated a vector of regression coefficients, *β*, from either a uniform distribution or a normal distribution. This represents the association between survival and gene expression.

(iii) Using the observations generated in (i) and the vector of regression coefficients *β *generated in (ii), we constructed a survival time from a Cox model with a specified baseline hazard function. Censoring times were generated from an exponential distribution with a parameter *λ*. The parameter *λ *was determined by the censoring fraction.

In the simulation study, we considered various parameters: the total number of genes (*p*), the sample size (*n*), the fraction of censoring (*c*_*p*_), the size of a gene set of interest (*m*), and the proportion of significant genes in the gene set (*m*_*p*_). To check the size of the five tests, we randomly generated gene expression variables from *MVN*(0,∑), ∑ = 0.2*I*_*p*_, where *I*_*p *_is an identity matrix of dimension *p *× *p*, and constructed survival times from a Cox model with *β*_*j *_= 0, *j *= 1,..., *p *and the constant baseline hazard rate of 0.005.

For the power calculation, we also randomly generated gene expression variables from *MVN*(0,∑) and constructed survival times from a Cox model with the parameter *β *and the baseline hazard function has an exponential distribution with the hazard rate of 0.005. Here ∑ and *β *were specified according to the following scenarios. First, we considered four different correlation structures of gene expression variables as implemented in Liu et al. [19] and Jung et al. [20]. Case (I) is that all gene expressions are independent, which assumes the correlation matrix as ∑ = (*σ*_*ij*_) with *σ*_*ii *_= 0.2 for *i *= 1,..., *p*; *σ*_*ij *_= 0 for *i *≠ *j*, with *i*, *j *= 1,..., *p. *Case (II) is that only significant genes are correlated within a gene set but non-significant genes are independent, that is, ∑ = (*σ*_*ij*_) with *σ*_*ii *_= 0.2 for *i *= 1,..., *p*; *σ*_*ij *_= 0.02 if two significant genes fall into the same gene set and *σ*_*ij *_= 0 otherwise. Case (III) is that there is an autoregressive correlation between significant genes, that is, ∑ = (*σ*_*ij*_) with *σ*_*ii *_= 0.2 for *i *= 1,..., *p*; *σ*_*ij *_*= *0.2 × 0.1^|^^*i*^^-^^*j*^^| ^if two significant genes fall into the same gene set and *σ*_*ij *_= 0 otherwise. Case (IV) is that there is an unstructured correlation between significant genes, that is, ∑ = (*σ*_*ij*_) with *σ*_*ii *_= 0.2 for *i *= 1,..., *p*; *σ*_*ij *_= 0.2 × *ρ*_*ij*_, if two significant genes fall into the same gene set and *σ*_*ij *_= 0 otherwise, where *ρ*_*ij *_is a random variable generated from *N*(0,0.1^2^). Secondly, we considered two different ways of generating the regression coefficient, *β*_*j*_, for *j *= 1,...,[*m *× *m*_*p*_], to investigate how the association of survival with genes affects the power for detecting the significant gene sets. Here [*a*] represents the greatest integer less than or equal to *a. *Case (A) is that the survival is positively associated with genes by generating only positive coefficients from a uniform distribution *U*(0.2,0.6). Case (B) is that survival is randomly associated with genes by generating the coefficient from *N*(0,0.5^2^). For the rest of the regression coefficients, we set *β*_*j *_= 0 for *j *= [*m *× *m*_*p*_]+1,..., *p*.

To assess the size and power of the tests, we implemented the simulation procedure as follows: (i) We calculated the test statistic for each method, (ii) permuted the samples 1000 times, recalculated the test statistics, and used these permuted test statistics to estimate the p-value, (iii) and then repeated procedures (i) and (ii) 500 times to estimate the size and 200 times to estimate the power, respectively. The size is estimated as the observed proportion of replications with a p-value smaller than the nominal size *α *= 0.05, and the power is estimated as the observed proportion of replications of in which the null hypothesis was correctly rejected at the nominal size *α *= 0.05.

Table 1 shows the sizes of the five tests for *p *= 200 with *n *= 50,80, *m *= 20,50, and *c*_*p *_= 0.0,0.1,0.3,0.5. As shown in Table 1, the empirical sizes of the five tests are well controlled across all possible combinations of parameters. Table 2 displays the power of the five tests for *p = *200, *n *= 80, *m *= 50 under the combinations of *c*_*p *_= 0.0,0.3 and *m*_*p *_*=*0.1,0.3,0.5 across all possible scenarios of gene correlation structure, and all scenarios of the association parameter.

**Table 1:** The estimated size of the five tests for *p *= 200 based on 500 iterations

*n*	*m*	*c*_*p*_	GSEA1	GSEA2	GT	WT	GBST
80	20	0.0	0.058	0.052	0.050	0.050	0.050
		0.1	0.060	0.052	0.058	0.058	0.062
		0.3	0.034	0.054	0.054	0.066	0.046
		0.5	0.062	0.042	0.040	0.032	0.052
	50	0.0	0.050	0.048	0.044	0.042	0.052
		0.1	0.050	0.056	0.058	0.058	0.036
		0.3	0.054	0.040	0.054	0.048	0.052
		0.5	0.068	0.060	0.058	0.058	0.060

50	20	0.0	0.054	0.042	0.036	0.032	0.044
		0.1	0.062	0.056	0.046	0.046	0.058
		0.3	0.056	0.036	0.044	0.038	0.052
		0.5	0.046	0.042	0.038	0.046	0.046
	50	0.0	0.070	0.046	0.052	0.044	0.046
		0.1	0.044	0.048	0.042	0.046	0.038
		0.3	0.050	0.038	0.060	0.058	0.060
		0.5	0.046	0.046	0.058	0.040	0.050

Note: *n *= Sample size; *m *= the size of gene set; *c*_*p *_= censoring proportion. GSEA1 = GSEA with the equal weight; GSEA2 = weighted GSEA; GT = Global Test; WT = Wald-type Test; GBST = Global Boost Test

**Table 2:** The estimated power of the five tests for *p *= 200, *n *= 80, and *m *= 50 based on 200 replications

			Case(A)					Case (B)				
			
Case	***c***_***p***_	***m***_***p***_	GSEA1	GSEA2	GT	WT	GBST	GSEA1	GSEA2	GT	WT	GBST
(I)	0.0	0.1	0.145	0.185	0.295	0.275	0.250	0.120	0.185	0.380	0.380	0.345
		0.3	0.290	0.370	0.650	0.630	0.635	0.250	0.355	0.750	0.745	0.805
		0.5	0.400	0.450	0.825	0.805	0.820	0.400	0.525	0.900	0.905	0.950
	0.3	0.1	0.145	0.155	0.155	0.165	0.155	0..65	0.110	0.240	0.270	0.265
		0.3	0.245	0.225	0.430	0.420	0.415	0.205	0.230	0.510	0.520	0.540
		0.5	0.335	0.330	0.615	0.605	0.620	0.275	0.395	0.725	0.695	0.765

(II)	0.0	0.1	0.165	0.225	0.425	0.485	0.320	0.075	0.135	0.330	0.320	0.295
		0.3	0.890	0.965	1.000	1.000	0.980	0.235	0.375	0.755	0.745	0.755
		0.5	1.000	1.000	1.000	1.000	1.000	0.430	0.605	0.900	0.890	0.925
	0.3	0.1	0.140	0.190	0.310	0.330	0.215	0.100	0.080	0.270	0.285	0.255
		0.3	0.730	0.815	0.955	0.970	0.880	0.215	0.295	0.520	0.520	0.570
		0.5	0.985	0.995	1.000	1.000	1.000	0.265	0.395	0.670	0.640	0.745

(III)	0.0	0.1	0.120	0.115	0.310	0.335	0.245	0.125	0.135	0.270	0.310	0.280
		0.3	0.355	0.460	0.790	0.765	0.735	0.270	0.375	0.745	0.725	0.805
		0.5	0.610	0.615	0.895	0.885	0.885	0.385	0.495	0.885	0.890	0.925
	0.3	0.1	0.085	0.095	0.215	0.195	0.190	0.070	0.075	0.215	0.220	0.225
		0.3	0.260	0.330	0.610	0.585	0.535	0.165	0.275	0.550	0.505	0.595
		0.5	0.390	0.445	0.710	0.700	0.725	0.305	0.350	0.735	0.740	0.810

(IV)	0.0	0.1	0.105	0.205	0.440	0.460	0.300	0.100	0.170	0.385	0.415	0.360
		0.3	0.815	0.885	0.990	0.990	0.965	0.255	0.340	0.755	0.755	0.760
		0.5	0.995	1.000	1.000	1.000	1.000	0.440	0.550	0.875	0.890	0.930
	0.3	0.1	0.160	0.215	0.305	0.310	0.265	0.135	0.165	0.240	0.255	0.235
		0.3	0.585	0.760	0.925	0.920	0.830	0.185	0.285	0.560	0.550	0.555
		0.5	0.960	0.970	1.000	1.000	0.985	0.320	0.410	0.680	0.685	0.760

Note: *m *= the size of gene set; *c*_*p *_= censoring proportion; *m*_*p *_= the proportion of significant genes in a gene set. GSEA1 = GSEA ith the equal weight; GSEA2 = weighted GSEA; GT = Global Test; WT = Wald-type Test; GBST = Global Boost Test. For Case (I), all genes are independent; for Case (II), there is within-correlation among significant genes; for Case (III), there is an autoregressive correlation between significant genes; and for Case (IV), there is an unstructured correlation between significant genes

**Figure 1:**
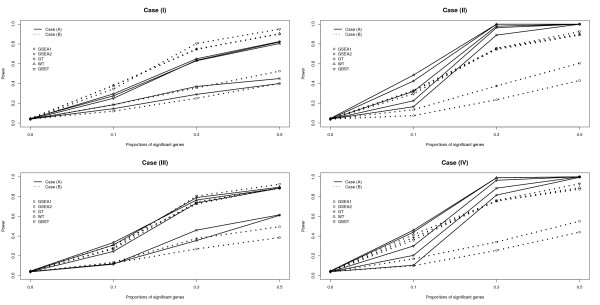
**The estimated size and power of the five tests, GSEA1, GSEA2, GT, WT and GBST, over the proportions of significant genes in each gene-set, using four different correlation structures, two different scenarios for generating the Cox regression coefficient vector, and censoring level *c*_*p *_= 0.0**. The solid and the dashed lines correspond to Case (A), that genes are positively associated with the survival times, and to the Case (B), that genes are randomly associated with the survival time, respectively. The circle, square, diamond, triangle and inverted triangle symbols correspond to GSEA1, GSEA2, GT, WT and GBST, respectively.

**Figure 2:**
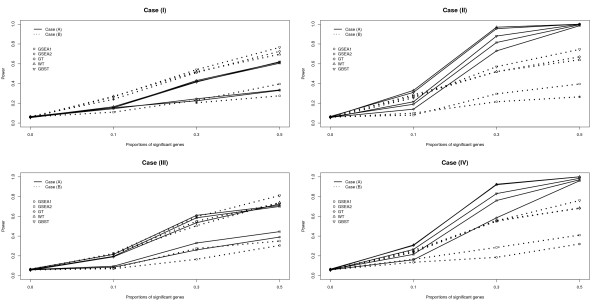
**Plots of estimated size and power of the five tests, GSEA1, GSEA2, GT, WT and GBST, over the proportions of significant genes in each gene-set, using four different correlation structures, two different scenarios for generating the Cox regression coefficient vector, and censoring level *c*_*p *_= 0.3**. The solid and dashed lines correspond to Case (A) that genes are positively associated with survival time, and to Case (B), that genes are randomly associated with the survival time, respectively. The circle, square, diamond, triangle and inverted triangle symbols correspond to GSEA1, GSEA2, GT, WT and GBST, respectively.

The results of Tables 1 and 2 are depicted in Figures 1 and 2 under the censoring fractions of *c*_*p *_= 0.0 and 0.3, respectively. In each plot, the ten different lines represent the power of the five tests under the two different scenarios of cases (A) and (B). The solid line represents the power of the five tests for Case (A) and the dotted line for Case (B). The power is plotted with *m*_*p*_, the proportion of the significant genes in each gene set, set to be 0.0,0.1,0.3 and 0.5. Here *m*_*p *_is considered to be the effect size because the proportion of significant genes affects the association between the survival time and the gene set. It is interesting that the power of the five tests differs depending on the combinations of the gene correlation structure and the association of genes with survival. For Case (I), the power of the five tests is higher for Case (B) than for Case (A). On the other hand, in the plots for cases (II) and (IV), the power of the five tests is much higher for Case (A) than for Case (B). This result implies that there might be a synergistic effect in the power of detecting significant genes when the genes are correlated and the survival is positively associated with genes. For Case (III), the power of GT, WT and GBST is almost the same regardless of the association of genes with survival, whereas both GSEA1 and GSEA2 have higher power for Case (A) than for Case (B). In general, as described in Figure 1, GT, WT and GBST consistently have higher power than GSEA1 and GSEA2. Comparing the two GSEA tests, the power of GSEA2 is equal to or slightly greater than the power of GSEA1, but these two tests have lower power than 0.5 except for the combinations of cases (II) and (IV) with Case (A) when *m*_*p *_≥ 0.3. As shown in Figure 2, the same pattern of power is found when the censoring fraction increases from *c*_*p *_= 0.0 to 0.3 though power consistently decreases as censoring increases.

### Analysis of a real example of ovarian cancer data

We next evaluated the performance of the five tests using an ovarian cancer data set from Dressman et al. [21]. This dataset consists of 119 ovarian cancer samples that were obtained at the initial cytoreductive surgery from patients treated at Duke University Medical Center and H. Lee Moffitt Cancer Center and Research Institute. 22115 gene expression levels were used in this analysis, of which 204 pathways were identified by KEGG. Among these 204 pathways, the smallest pathway consists of 5 genes while the largest one includes 474 genes, and the average number of genes across the 204 pathways is about 80.

Crijns et al. [22] identified survival-related profile, pathways and transcription factors by analyzing a dataset of 157 patients with advanced-stage serious ovarian cancer from the University Medical Center Groningen in Netherlands, collected in the time period 1990-2003. They used the dataset of 119 ovarian cancer samples in Dressman et al. [21] to validate the identified profile and pathways.

We compared the pathways identified by the five tests with those reported in Dressman et al. [21] and Crijns et al. [22]. In Dressman et al. [21], the significant pathways were found by a binary logistic regression model analysis and a stochastic regression model search, called shotgun stochastic search. On the other hand, Crijns et al. [22] identified the significant pathways using the functional class scoring analysis, in which the p-value of a univariate Cox proportional hazards is computed for all genes, and then p-values of each pathway are summarized by the mean negative logarithm of single gene p-values (LS statistic), as well as the Kolmogorov-Smirnov (KS) statistic for testing if the p-values come from a uniform distribution. The significance of each pathway is evaluated by computing the empirical distribution of these summary statistics in random samples of genes.

Table 3 displays 19 gene sets with an FDR threshold of *q *< 0.1 (Benjamini and Hochberg [23]) based on at least one of the five tests, along with the corresponding univariate p-value. The underlined pathways were also reported to be significant in Dressman et al. [21] and Crijns et al. [22]. As shown in Table 3, GT, WT and GBST have greater power to detect significant gene sets than GSEA1 and GSEA2. For example, GT, WT and GBST detect 12, 6 and 8 significant pathways among 204 pathways, respectively, while none of pathways is identified by either GSEA1 or GSEA2. Crijns et al. [22] listed 17 pathways in their study, of which 16 were confirmed by Dressman et al. [21]. Among those, with an FDR threshold of *q *< 0.1, three pathways were identified by at least one of GT, WT and GBST. GT identified all three pathways - Purine metabolism, Leukocyte transendothelial migration and Jak-STAT signaling - and GBST identified only one pathway, Leukocyte transendothelial migration. None of the pathways were identified by WT.

**Table 3:** Pathways with more genes associated with overall survival as identified by either GT or WT at the nominal 0.01 level of the permutation test

Pathway name	Gene set size	p-value				q-value			
		
		GSEA1	GSEA2	GT	WT GBST	GSEA1	GSEA2	GT	WT GBST
Pentose phosphate pathway	39	0.010	0.003	0.000	0.000 0.003	0.921	0.612	0.000	0.000 0.087

Histidine metabolism	54	0.070	0.012	0.000	0.000 0.000	0.921	0.638	0.000	0.000 0.000

Tryptophan metabolism	86	0.034	0.024	0.002	0.003 0.009	0.921	0.701	0.061	0.085 0.140

One carbon pool by folate	28	0.065	0.027	0.010	0.000 0.010	0.921	0.701	0.108	0.000 0.140

DNA replication	52	0.021	0.013	0.009	0.002 0.037	0.921	0.638	0.108	0.077 0.207

Colorectal cancer	165	0.139	0.145	0.003	0.010 0.000	0.921	0.811	0.073	0.136 0.000

Nucleotide excision repair	56	0.047	0.068	0.014	0.003 0.054	0.921	0.811	0.129	0.085 0.253

Urea cycle and metabolism of amino groups	40	0.031	0.259	0.010	0.036 0.003	0.921	0.878	0.108	0.191 0.087

Purine metabolism	201	0.081	0.026	0.003	0.009 0.072	0.921	0.701	0.074	0.136 0.275

Aminophosphonate metabolism	21	0.267	0.028	0.000	0.009 0.056	0.921	0.701	0.000	0.136 0.253

Pantothenate and CoA biosynthesis	19	0.252	0.061	0.189	0.010 0.003	0.921	0.811	0.337	0.136 0.087

Leukocyte transendothelial migration	197	0.332	0.161	0.004	0.013 0.003	0.921	0.811	0.074	0.166 0.087

Inositol metabolism	7	0.234	0.274	0.035	0.044 0.004	0.921	0.878	0.207	0.204 0.089

Tyrosine metabolism	81	0.531	0.147	0.017	0.018 0.003	0.970	0.811	0.133	0.171 0.087

Lysine degradation	56	0.124	0.225	0.004	0.018 0.137	0.921	0.878	0.074	0.171 0.362

Starch and sucrose metabolism	72	0.373	0.133	0.002	0.033 0.250	0.928	0.811	0.068	0.191 0.427

Glycerophospholipid metabolism	89	0.535	0.255	0.004	0.028 0.036	0.970	0.878	0.074	0.190 0.207

Androgen and estrogen metabolism	53	0.386	0.168	0.005	0.035 0.147	0.935	0.811	0.085	0.191 0.372

Jak-STAT signaling pathway	240	0.258	0.606	0.001	0.036 0.121	0.921	0.892	0.051	0.191 0.358

Starch and sucrose metabolism	72	0.373	0.133	0.002	0.033 0.250	0.928	0.811	0.068	0.191 0.427

Note: The underlined pathways are identified by Crijns et al.(2009).

## Discussion

Many gene-set analysis proposals have been compared in simulation studies and on real data sets. However, most studies have dealt with a binary phenotype like the presence or absence of disease, or treatment vs. control. In this paper, we focused on the survival phenotype and compared five different gene-set analysis tests, GSEA1, GSEA2, GT, WT and GBST, in a simulation study and on two ovarian cancer data sets. From the simulation results, we found that GT, WT, and GBST are more powerful than GSEA1 and GSEA2. Furthermore, the power of the five tests is substantially affected by the correlation structure of genes and the association between survival and the genes. The power of the five tests can approach 1.0 when the genes are correlated, survival is positively associated with the gene expression values, and *m*_*p *_≥ 0.3. For more powerful test results, it might be desirable to check the correlation structure among genes within each pathway and the direction of association between genes and survival.

Although GSEA was originally based on the rank of the correlation coefficients between a binary phenotype and gene expression levels, we replaced the correlation coefficient by the regression coefficient from a Cox model and took the rank of the absolute value of its standardized coefficient. We then followed the rest of the GSEA procedure in order to identify significant gene-sets or pathways. This is a modification of GSEA that takes into account the survival phenotype, which may in some cases be more informative than a binary phenotype. In addition, the modified GSEA allows covariance adjustment for age, sex and other clinical variables by taking the regression coefficient of genes from the Cox model with those adjusting covariates. However, the performance of the GSEA tests, GSEA1 and GSEA2, are not satisfactory except for a few cases. This may be due to the fact that the GSEA tests are nonparametric approaches using the rank-based statistic instead of using the value of the regression coefficient.

On the other hand, GT, WT and GBST have been proposed for regression-based models and can be extended to any phenotype such as binary, continuous, multi-class, or survival. These three tests can be easily adjusted for covariates, in order to determine whether gene expression profiles have an association with survival beyond what is explained by the adjusting covariates. The GT method assumes a random-effect model for the parameters corresponding to the coefficients of the individual genes in the pathway, in which the parameters are random variables and samples from *N*(0,τ^2^). Here all parameters are assumed to have a common variance of *τ*^2^, which can be extended to have a more complex covariance structure as mentioned in Goeman et al. [6]. The GT method uses a score test of *τ*^2 ^= 0, which is equivalent to the null hypothesis that there is no association between survival and a given set of genes. Therefore, this test does not depend on the number of genes in a given set and works under the assumption of a random-effect model with a common variance. As discussed in Goeman et al. [6], the score test of GT can have the optimal power against alternatives with small values of parameter *τ*^2^. In the simulation study, we generated the parameter from either a uniform or normal distribution with small variances. This may have contributed to the high power that we observed for GT. On the other hand, Boulesteix and Hothorn [8] proposed the GBST for testing the additional predictive value of gene expression data while adjusting for clinical covariates. They combined the standard regression models such as a logistic regression or a Cox model with a boosting procedure and used a permutation-based testing scheme. While Goeman et al. [5] reduced the multi-dimensional gene profiles into one-dimensional variance using a random-effect model, Adewale et al. [9] instead computed the sum of squares of the Wald statistics for the genes in the pathway from the univariate regression model. Likewise, the WT does not depend on the multi-dimensionality of the gene sets since the test statistic can be summarized as the sum of individual association measures. Compared to the GSEA tests, these three tests are based on parametric approaches since the values of the regression coefficients are taken into account in these tests via a score statistic or a sum of squares.

As pointed out by a referee, methodological issues should be considered when comparing gene-set analysis methods. Goeman and Bühlmann [24] addressed the definition of the null hypothesis, and in particular competitive versus self-contained tests, as well as the calculation of p-values, and the use of gene sampling versus subject sampling methods. GT, WT and GBST are based on the classical statistical models which lead to test a self-contained null hypothesis. Furthermore, they calculate the p-value using a subject sampling model. On the other hand, GSEA is a hybrid method: a Kolmogorov-Smirnov test statistic is motivated by a gene-sampling model, whereas a subject-sampling model is used to calculate the p-value. It was also pointed out that GSEA sometimes shows low power since the model and null hypothesis used to motivate the test statistic are different from the model and null hypothesis used to calculate the p-value. Simulation results indicate that both GSEA1 and GSEA2 have lower power than GT, WT and GBST.

One of the important advantages of gene-set analysis over the single gene approach is that the multiple testing problems can be alleviated because the number of pathways is much smaller than that of genes. In the ovarian cancer example, for example, there are 22115 genes. The number of pathways is 204, which is more than a 100-fold reduction. When a single pathway is of interest in pathway analysis, the issue of multiple testing is not a problem. However, when multiple pathways are of interest, as in cases when pathway analysis is exploratory with many pathways of potential interest, then multiple comparison methods such as false-discovery rates (FDR) must be applied. In the ovarian cancer example, we used FDR to control 204 pathway comparisons. A few pathways were found to have *q *< 0.1. It is noted that all 17 pathways identified by Crijns et al. [22] contain more than 150 genes. Their sizes vary from 150 to 474, and their median is 239. However, GT detects 12 pathways whose sizes range from 39 to 240 with a median of 79, WT detects 6 pathways whose sizes range from 28 to 86 with a median of 53, and GBST detects 8 pathways whose sizes range from 7 to 165 with a median of 47. In summary, GT, WT and GBST detected pathways with a variety of gene-set sizes, whereas Crijns et al. [22] detected only large gene sets.

To implement GT and GBST, we adapted the function 'gt' in the R package 'globaltest', and the function 'globalboosttest' in the R package 'globalboosttest'. We implemented GSEA1, GSEA2, and WT from scratch in R, using the function 'coxph'. For the readers' convenience, all codes are available as additional files. The additional file 1 is R source codes for generating data, calling subroutines, and calculating permuted p-values and the additional file 2 is R source codes for calculating five test statistics, GSEA1, GSEA2, GT, WT, and GBST.

## Conclusion

In conclusion, GT, WT and GBST have high power to identify gene sets whose expression is associated with survival. Survival is often strongly affected by well-known clinical variables such as stage, type of tumor, and tumor size, as well as demographic variables such as age, sex, and race. Therefore, it is very important to evaluate the effects of genes on survival while considering known covariates. Since these tests allow for adjustment for covariates, they assess whether expression of a given set of genes has an association with survival after controlling for confounders. In general, GT, WT and GBST are more powerful than GSEA1 and GSEA2. However, when genes are correlated within each pathway and survival is positively associated with genes, it does not matter which test is applied to detect significant gene sets, provided that the number of significant genes is moderately large within each pathway.

## Methods

In this paper, we compared four gene-set analysis methods, GSEA, Global Test, Wald-type Test and Global Boost Test, when the phenotype of interest is survival. These methods involve the test statistic from a Cox model to predict survival gene expression. The Cox model assumes that the hazard function at time *t *is specified as a function of covariates as follows:

$$h\left(t\right)=h_{0}\left(t\right)\exp\left(\beta^{\prime }X\right),$$

where *h*_0_(*t*) is a baseline hazard function, *X *= (*x*_1_,..., *x*_*p*_)' is the *p*-dimensional covariate vector, and *β *is a *p *× 1 regression coefficient vector relating to gene expression.

### (1) GSEA

Let *S *be an a priori defined set of genes, out of a total of *p *genes in a microarray dataset. GSEA tests the null hypothesis that the expression of the genes in *S *is not associated with a phenotype of interest. The procedure is as follows:

(i) Compute a correlation or association measure between each of the *p *genes and phenotype. Here we used a *t*-test statistic, *r*_*j *_= *b*_*j*_/*s*_*j*_, *j *= 1,..., *p*, where *b*_*j *_is the parameter estimate of the log-hazard ratio, *β*_*j*_, for the *j*th gene's association with survival, and *s*_*j *_is its corresponding standard error.

(ii) Order the *p *genes by the absolute values of *r*_*j*_, from largest to smallest.

(iii) Compute the Enrichment Score (ES) as follows: start with *ES *= 0 and sum up from the top rank (*j *= 1) to the last rank (*j *= *p*), increasing by $|r_{j}{|}^{w}∕\underset{k\in S}{\sum }|r_{k}{|}^{w}$ if the *j*th gene belongs to the gene set *S*, and decreasing by 1/(*p*-*m*), otherwise, where *w *∈ [0,1] and *m *is the number of genes in the set *S*.

(iv) Take the maximum deviation from zero of the *ES *values among the *p *genes as the test statistic for the gene set *S.*

(v) Randomly permute the survival times and repeat (i)-(iv) many times.

(vi) Compute the significance level by comparing the observed value of the test statistic from (iv) to its permutation distribution obtained from (v).

We considered two different GSEA tests by setting *w *= 0,1, respectively. For *w *= 0, the resulting GSEA test (GSEA1) is a normalized Kolmogorov-Smirnov statistic, which is the original GSEA proposal of Mootha et al. [1]. However, it was shown by Subramanian et al. [2] that the original GSEA with *w *= 0 may have high *ES *scores for a set clustered near the middle of the ranked list, even though such a gene set likely is not truly associated with the phenotype. To address this issue, weighting the test statistic according to the correlation between each gene and the phenotype was proposed by Subramanian et al. [2]. For *w *= 1, we are weighting the genes in *S *by their correlation with the phenotype normalized by the sum of the correlation over all the genes in *S*. The main idea of the weighted GSEA (GSEA2) is to allow the magnitude of the increase in *ES *to reflect the correlation between the gene and the phenotype. In other words, the more highly a gene is correlated with the phenotype, the larger the increase in *ES *that gene is assigned. By comparing the performance of GSEA1 and GSEA2, the effect of weighting on the power can be investigated in simulation studies.

### (2) Global Test

The Global Test is also based on the regression coefficient from a Cox model. It tests the null hypothesis that all regression coefficients between survival and gene expression are zero, as follows:

$$H_{0}:\beta_{1}=\beta_{2}=\cdots =\beta_{m}=0.$$

We assume that all regression coefficients are random and sampled from a common distribution with mean zero and common variance *τ*^2^. Then the null hypothesis of no differential gene expression is reduced to the hypothesis that *τ*^2 ^= 0. The Global Test is a score test based on random-effect modeling of parameters corresponding to the coefficients of the individual genes in the pathway. Goeman et al. [5] originally proposed the Global Test based on the generalized linear model and then extended it to survival in the Cox model, in which a score test was derived for testing the null hypothesis *τ*^2 ^= 0 based on the martingale residual. The test statistic is given as follows:

$$Q=\frac{T-\hat{E}T}{\hat{V}ar(T)}.$$

Here, *T *= (*d *- *û*)' *R(d-û) - trace(RÛ) *is a function of the martingale residual, *d *- *û*, *R *= *XX*', and *Û = diag(û)*, where *X *is a *n *× *m *design matrix representing *m *gene expressions for *n *individuals, and *û = *(*û*_1_, *û*_2_,..., *û*_*n*_)' is the *n *× 1 vector of the estimated cumulative hazard function for the *i*th individual up to time *t_i_*.

### (3) Wald-type Test

This test is based on the unified pathway method proposed by Adewale et al. [9], which combines component-wise test statistics for significance of a subset of genes or pathway. The form of the test statistic is a sum of squares of the Wald statistic for individual genes constituting the pathway, denoted as follows:

$$W=\overset{m}{\underset{j=1}{\sum }}r_{j}^{2}.$$

### (4) Global Boost Test

This test is a permutation-based testing procedure to globally assess the additional predictive power of the expression values of a large number of genes, while adjusting for a few clinical covariates. Boulesteix and Hothorn [8] combined two well-known statistical tools, Cox regression and a boosting algorithm. The procedure of GBST originally consists of two steps: in the first step, the effects of clinical variables are estimated and the corresponding linear predictor is used as an offset in the second step. However, only intercept was used as an offset in this simulation study because any clinical variables were not considered for the comparable result among five tests. As a result, the procedure of GBST begins with running the boosting algorithm given in the section 'boosting with componentwise linear least squares' in Boulesteix and Hothorn [8] using the partial log-likelihood function. Through the number of boosting iterations, GBST derived the resulting linear predictor as ${\hat{\eta }}_{i}^{}={\hat{\beta }}_{0}+{\hat{\beta }}_{1}x_{i1}+\cdots +{\hat{\beta }}_{m}x_{im}$ by minimizing the average negative partial log-likelihood for a Cox model. Here the partial log-likelihood function is given as follows:

$$l\left(\beta \right)=\overset{n}{\underset{i=1}{\sum }}\delta_{i}\left(\eta_{i}-\log\left(\overset{n}{\underset{j=1}{\sum }}I\left(t_{j}\geq t_{i}\right)\exp\left(\eta_{j}\right)\right)\right)\;,$$

where *δ*_*i *_is equal to 1 if an event occurred at that time and 0 if the observation has been censored, and *I*(·) is an indicator function taking 1 if its argument is true, i.e., if individual *j *is still under risk just before time *t*_*i*_, and value 0 otherwise.

## Authors' contributions

SYL conceived the study and drafted the manuscript, JHK designed the simulation study and SHL participated in the design of the study and performed the statistical analysis. All authors read and approved the final manuscript

## Supplementary Material

Additional file 1 **R source codes for generating data, calling subroutines, and calculating permuted p-values**. This is a main program for generating simulated data sets and calling a subroutine of calculating test statistics. It also performs to generate the permuted samples with a given simulated data repeatedly and calculates the permuted p-value of each test comparing the observed test statistic with the distribution of the permuted test statistics. Click here for file

Additional file 2 **R source codes for calculating five test statistics, GSEA1, GSEA2, GT, WT, and GBST**. This is a subroutine for calculating five test statistics included in the article such as GSEA, Global Test, Wald-type Test, and Global Boost Test. Click here for file
